# Real-time sleep disorder monitoring design using dynamic temporal graphs with facial and acoustic feature fusion

**DOI:** 10.3389/frai.2025.1681759

**Published:** 2025-11-11

**Authors:** Fei Pei, Ying Zhou, Qiangqiang Fu, Hong Zhou

**Affiliations:** 1 Department of Otolaryngology, Shidong Hospital, Shanghai, China; 2 Department of Geriatrics, Shidong Hospital, Shanghai, China; 3 Yangpu Hospital, School of Medicine, Tongji University, Shanghai, China

**Keywords:** sleep disorder detection, facial expression analysis, real-time health monitoring, multimodal learning, machine learning

## Abstract

**Introduction:**

Sleep disorders pose significant risks to patient safety, yet traditional polysomnography imposes substantial discomfort and laboratory constraints. We developed a non-invasive multimodal monitoring system for real-time sleep pathology detection.

**Methods:**

We integrated facial expression analysis via deep convolutional neural networks with audio signal processing for breathing pattern detection. Heterogeneous data streams were unified into dynamic graph representations, with graph neural networks modeling spatiotemporal patterns of sleep pathologies.

**Results:**

The system accurately detected sleep apnea, restless leg syndrome, and cardiovascular irregularities with 10.7-s average delay and 94.6% clinical agreement, achieving diagnostic accuracy comparable to polysomnography.

**Conclusion:**

This framework enables continuous non-invasive monitoring for point-of-care screening and home-based management, potentially expanding sleep medicine access for underserved populations.

## 1 Introduction

Sleep disorders affect millions of people worldwide and represent a significant public health concern, with conditions such as sleep apnea, insomnia, and parasomnias contributing to increased morbidity, reduced quality of life, and elevated healthcare costs (Alshammari, 2024; Yildirim et al., 2019; Sharma et al., 2021b). The accurate detection and monitoring of sleep-related pathological conditions is crucial for timely medical intervention and prevention of serious complications (Morokuma et al., 2023; Arslan et al., 2023). Traditional sleep monitoring approaches, primarily relying on polysomnography (PSG) in controlled laboratory environments, while considered the gold standard, are expensive, time-consuming, and often impractical for long-term monitoring or home-based care (Ha et al., 2023; Brink-Kjaer et al., 2022). Moreover, PSG requires multiple electrodes and sensors that can disturb patients' natural sleep patterns, potentially affecting the reliability of diagnostic outcomes (Rahman et al., 2025; Reis et al., 2024).

Recent advances in wearable technology and non-invasive monitoring systems have opened new avenues for sleep assessment. Current approaches predominantly focus on single-modality solutions, such as actigraphy for movement detection, heart rate variability analysis for autonomic nervous system assessment, or audio-based detection of breathing irregularities (Hussain et al., 2022; Yoon and Choi, 2023). However, these unimodal approaches suffer from several critical limitations. First, they often lack the comprehensive information necessary to capture the complex, multifaceted nature of sleep disorders, which typically manifest through various physiological and behavioral indicators simultaneously (Nguyen et al., 2023). Second, single-modality systems are susceptible to noise, artifacts, and environmental interference (Boiko et al., 2023), leading to reduced accuracy and reliability in real-world deployment scenarios.

Facial expression analysis has emerged as a promising non-invasive approach for detecting physiological states and emotional conditions during sleep (Maranci et al., 2021; Huang et al., 2023). Research has demonstrated that facial expressions can provide valuable insights into pain levels, breathing difficulties, and neurological activities during sleep. Similarly, audio signal analysis has shown significant potential in detecting sleep apnea events, snoring patterns, and other respiratory irregularities (Rosamaria et al., 2023; Xu et al., 2020). However, existing studies have primarily treated these modalities independently (Lv et al., 2020), failing to leverage their complementary information and temporal correlations.

The integration of multimodal data for sleep monitoring presents several fundamental challenges (Wang et al., 2025b). First, different modalities operate at varying temporal scales and exhibit distinct data characteristics, making it difficult to establish meaningful correlations and extract unified representations (Cheng et al., 2023; Torres et al., 2018). Facial expressions may change subtly over minutes, while audio signals contain high-frequency components that vary within seconds. Second, the temporal dependencies within and across modalities are complex and non-linear (Zhai et al., 2020; Zahid et al., 2023), requiring sophisticated modeling approaches that can capture both short-term fluctuations and long-term trends. Third, sleep disorders often manifest through subtle, gradual changes that may not be immediately apparent in individual modalities but become significant when considered collectively over extended periods (Duan et al., 2021; Lin et al., 2023). Existing multimodal fusion techniques, while successful in other domains, face specific challenges when applied to sleep monitoring (Liao et al., 2024). Traditional early fusion approaches that concatenate features from different modalities often result in high-dimensional representations that are prone to overfitting and computational inefficiency. Late fusion methods that combine decisions from individual modality classifiers may miss important cross-modal interactions (Zhai et al., 2021) that are crucial for accurate sleep disorder detection. Furthermore, most current approaches treat sleep monitoring as a static classification problem (Chung et al., 2017), ignoring the inherently dynamic and temporal nature of sleep processes.

**Figure 1:**
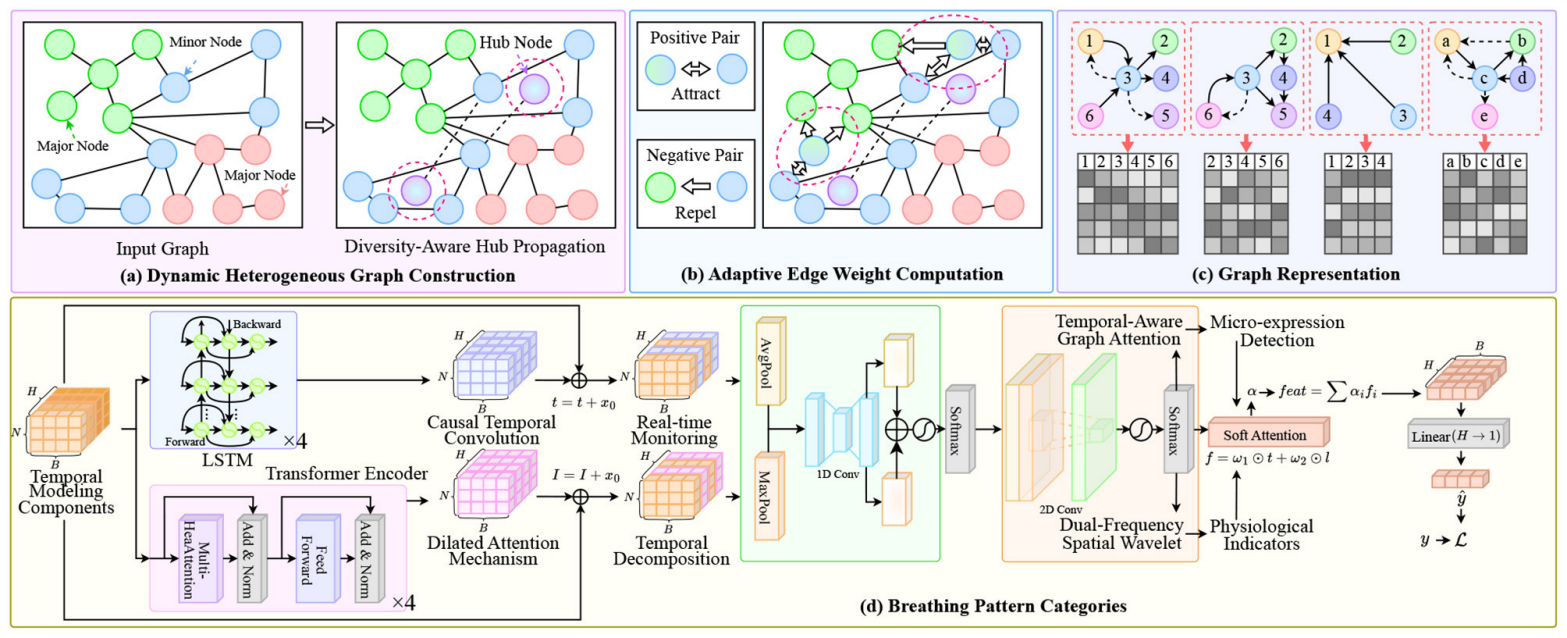
Overview of our multimodal dynamic graph network framework for sleep disorder monitoring. The system processes multimodal inputs through: **(A)** Dynamic heterogeneous graph construction with diversity-aware hub propagation to balance information flow across facial and audio modalities; **(B)** Adaptive edge weight computation using positive/negative pair attraction-repulsion mechanisms to enhance cross-modal alignment; **(C)** Graph representation encoding with temporal-aware attention for structural pattern learning; **(D)** Breathing pattern categorization module integrating LSTM-based temporal modeling, causal convolution for real-time monitoring, dilated attention mechanism for long-range dependencies, dual-frequency spatial wavelet analysis, and micro-expression detection for physiological indicators.

To address these limitations, we propose a novel multimodal dynamic graph neural network framework that integrates facial expression analysis and sleep audio signal processing for real-time detection and prediction of sleep-related pathological conditions in Figure 1. Our approach is built upon several key insights and innovations. First, we conceptualize the multimodal sleep monitoring problem as a dynamic graph learning task, where **different modalities and their temporal states are represented as nodes in a time-evolving graph structure**. This representation naturally captures the heterogeneous nature of multimodal data while preserving the temporal dependencies crucial for understanding sleep dynamics. Nodes in our graph represent feature vectors extracted from facial expressions and audio signals at different time points, while edges encode both intra-modal temporal relationships and inter-modal correlations. Second, we develop a specialized graph neural network architecture that can effectively learn from this dynamic multimodal graph representation. Our model incorporates attention mechanisms to **automatically weight the importance of different modalities and temporal segments**, allowing the system to focus on the most relevant information for detecting specific sleep disorders. The architecture includes dedicated modules for processing facial expression data using convolutional neural networks optimized for low-light sleep environments, and audio processing components that can handle various acoustic patterns associated with different sleep pathologies. Third, we introduce a temporal modeling component that explicitly **captures the evolution of sleep states over time**. Unlike traditional approaches that analyze fixed time windows independently, our framework maintains a continuous representation of the patient's sleep state that evolves dynamically as new data becomes available. This enables early detection of developing conditions and provides predictive capabilities for anticipating potential sleep-related medical events.

Our technical approach consists of several interconnected components designed to address the specific challenges of multimodal sleep monitoring. The facial expression analysis module utilizes lightweight convolutional neural networks optimized for processing infrared or low-light facial images captured during sleep. We employ specialized preprocessing techniques to handle variations in lighting conditions, head pose changes, and occlusions commonly encountered in sleep environments. Feature extraction focuses on detecting micro-expressions and subtle facial movements that may indicate discomfort, breathing difficulties, or neurological activities. The audio processing component employs advanced signal processing techniques to extract meaningful features from sleep audio recordings. This includes spectral analysis for detecting breathing patterns, time-frequency analysis for identifying apnea events, and novel acoustic feature extraction methods for recognizing various sleep-related sounds. We address challenges related to background noise, signal variability across different recording devices, and the need for real-time processing in resource-constrained environments. The dynamic graph construction mechanism creates time-evolving graph representations that capture the complex relationships between different modalities and their temporal evolution. We develop novel graph edge weighting schemes that automatically adapt based on the reliability and relevance of different modalities at different time points. This adaptive approach ensures robust performance even when individual modalities are compromised by noise or artifacts. Our graph neural network architecture incorporates several innovative components, including multi-scale temporal attention mechanisms, cross-modal correlation modules, and specialized pooling operations designed for handling irregular time series data. The model is trained using a combination of supervised learning for known sleep disorder patterns and self-supervised learning techniques that leverage the inherent structure of multimodal sleep data.

The proposed framework offers several significant advantages over existing approaches. By leveraging the complementary information from multiple modalities, our system can achieve higher accuracy and robustness compared to single-modality solutions. The dynamic graph representation enables the capture of complex temporal patterns that are crucial for understanding sleep disorders, while the attention mechanisms provide interpretability by highlighting the most relevant features and time periods for specific predictions. This research contributes to the growing field of multimodal health monitoring by providing a novel framework that can effectively integrate heterogeneous data sources for complex medical applications. Our work advances the state-of-the-art in both multimodal learning and sleep medicine, offering new possibilities for personalized and continuous healthcare monitoring solutions.

## 2 Methods

Let us formally define the multimodal sleep monitoring problem as a dynamic graph learning task. We denote the multimodal sleep data as a collection D={F,A}, where $F={\left\{f_{t}\right\}}_{t=1}^{T}$ represents the sequence of facial expression features and $A={\left\{a_{t}\right\}}_{t=1}^{T}$ represents the corresponding audio signal features over time horizon *T*. At each time step *t*, we have $f_{t}\in {\mathbb{R}}^{d_{f}}\;\text{(facial features)}$ and $a_{t}\in {\mathbb{R}}^{d_{a}}\;\text{(audio features)}$, where *d*_*f*_ and *d*_*a*_ are the dimensionalities of facial and audio feature spaces, respectively in Table 1. The objective is to learn a mapping function M:D→Y that predicts sleep pathology labels $y_{t}\in Y=\left\{0,1,2,...,K\right\}$ at each time step, where *K* represents the number of distinct sleep disorder categories.

**Table 1:** Mathematical notation and symbols used in methods section.

**Symbol**	**Description**	**Symbol**	**Description**
D	Multimodal sleep data collection	*G*_*t*_	Dynamic heterogeneous graph at time *t*
F	Facial expression feature sequence	$V_{t}$	Node set containing facial and audio nodes
A	Audio signal feature sequence	$E_{t}$	Edge set for intra- and cross-modal connections
*f*_*t*_	Facial features at time *t*	*X*_*t*_	Node feature matrix at time *t*
*a*_*t*_	Audio features at time *t*	*d*_*f*_, *d*_*a*_	Dimensionalities of facial and audio features
*y*_*t*_	Sleep pathology labels at time *t*	*K*	Number of sleep disorder categories
*T*	Time horizon	M	Mapping function for prediction
*I*_*t*_	Input facial image at time *t*	*H, W, C*	Image height, width, and channels
*X* ^(*l*)^	Feature maps at layer *l*	*W* ^(*l*)^	Learnable parameters at layer *l*
$f_{t}^{raw}$	Raw facial features before attention	*A*_*spatial*_	Spatial attention mechanism
*A*_*temporal*_	Temporal attention mechanism	*Q, K, V*	Query, key, and value matrices
*h*_*t*−1_	Hidden state from previous time step	*W*_*t*_, *W*_*f*_, *W*_*h*_	Learnable weight matrices
*S*_*t*_	Short-Time Fourier Transform at time *t*	ψ_*j, k*_	Mother wavelet at scale *j*, position *k*
*M*_*t*_	Power Spectral Density	*C*_*t*_	Cepstral coefficients
ZCR_*t*_	Zero Crossing Rate	RMS_*t*_	Root Mean Square energy
SC_*t*_	Spectral Centroid	SRO_*t*_	Spectral Rolloff
*W*_1:*J, t*_	Wavelet coefficients	*N*	Number of samples
$x_{t}^{f},x_{t}^{a}$	Projected facial and audio node features	*W*_*f*_, *W*_*a*_	Projection matrices
$\alpha_{ij}^{temp}$	Temporal edge attention weight	$\alpha_{ij}^{cross}$	Cross-modal edge attention weight
*w*_*ij*_	Final edge weight	λ_1_, λ_2_, λ_3_	Hyperparameters
γ	Temporal decay rate	$N_{i}$	Neighborhood of node *i*
*H* ^(*l*)^	Hidden representations at layer *l*	$A_{s}^{\left(l\right)}$	Adjacency matrix at scale *s*
*S*	Number of temporal scales	*D*	Degree matrix
$e_{ij}^{\left(l\right)}$	Attention energy between nodes *i, j*	$\alpha_{ij}^{\left(l\right)}$	Attention coefficient
ϕ(*t*_*i*_, *t*_*j*_)	Temporal relationship encoding	ω_*d*_	Frequency parameters
$h_{f}^{\left(L\right)},h_{a}^{\left(L\right)}$	Final layer facial and audio features	*Q*_*f*_, *K*_*a*_, *V*_*a*_	Cross-modal attention components
Attn_*f*→*a*_	Facial-to-audio attention	Attn_*a*→*f*_	Audio-to-facial attention
*h*_*fused*_	Fused multimodal representation	*d*_*k*_	Key dimension
*r*_*t*_, *z*_*t*_	Reset and update gates in GRU	${\tilde{s}}_{t}$	Candidate hidden state
*s*_*t*_	Final hidden state	*U*_*r*_, *U*_*z*_, *U*_*s*_	Recurrent weight matrices
$s_{t}^{\left(\ell \right)}$	Multi-scale decomposition at level ℓ	*K*_ℓ_	Number of wavelets at level ℓ
$\alpha_{k}^{\left(\ell \right)}$	Learnable wavelet coefficients	ϕ	Mother wavelet function
$h_{t}^{\left(c\right)}$	Causal convolution output	*k*	Kernel size
*d*	Dilation factor	*M*_*causal*_	Causal attention mask
*R*	Attention radius	*W*_*pos*_	Positional encoding weights
$L_{cls}$	Classification loss	$L_{temp}$	Temporal consistency loss
$L_{cont}$	Contrastive loss	$L_{rec}$	Reconstruction loss
α_*k*_	Class-specific weights	γ	Focusing parameter
ŷ_*t, k*_	Predicted probability for class *k*	ω_*t*_	Adaptive temporal weight
β	Similarity threshold parameter	τ	Temperature parameter
η_*t*_	Learning rate at time *t*	η_*min*_, η_*max*_	Minimum and maximum learning rates
*T*_*cur*_	Current epoch in restart cycle	*T*_*i*_	Epochs in restart cycle

### 2.1 Facial expression feature extraction

For facial expression analysis, we employ a modified ResNeXt-50 architecture with specialized attention mechanisms for low-light sleep environments. The facial feature extraction process can be formulated as $X^{\left(0\right)}=\text{Preprocess}\left(I_{t}\right)$, $X^{\left(l+1\right)}=F_{\text{ResNeXt}}^{\left(l\right)}\left(X^{\left(l\right)},W^{\left(l\right)}\right)$ and $f_{t}^{\text{raw}}=\text{GlobalAvgPool}\left(X^{\left(L\right)}\right)$, where $I_{t}\in {\mathbb{R}}^{H\times W\times C}$ represents the input facial image at time *t*, *X*^(*l*)^ denotes the feature maps at layer *l*, and *W*^(*l*)^ are the learnable parameters (Yang et al., 2021). To enhance the feature representation for sleep-specific facial expressions, we introduce a temporal-spatial attention mechanism $A_{\text{spatial}}=\text{softmax}\left(\frac{QK^{T}}{\sqrt{d_{k}}}\right)$:


$$\begin{array}{l} A_{\text{temporal}}=\text{softmax}\left(W_{t}\tanh\left(W_{f}f_{t}^{\text{raw}}+W_{h}h_{t-1}\right)\right) \end{array}$$ (1)

$$\begin{array}{l} f_{t}=A_{\text{temporal}}⊙A_{\text{spatial}}V, \end{array}$$ (2)

where *Q*, *K*, *V* are query, key, and value matrices, *W*_*t*_, *W*_*f*_, *W*_*h*_ are learnable weight matrices, *h*_*t*−1_ is the hidden state from the previous time step, and ⊙ denotes element-wise multiplication.

### 2.2 Audio signal feature extraction

For audio signal processing, we implement a multi-scale wavelet transform combined with spectral analysis. The audio feature extraction pipeline is defined as $S_{t}=\text{STFT}\left(a_{t}^{\text{raw}}\right),$ $W_{j,k}=\underset{n}{\sum }a_{t}^{\text{raw}}\left[n\right]\psi_{j,k}^{*}\left[n-k\right],$ $M_{t}=|S_{t}{|}^{2}\;\text{(Power Spectral Density)},$ and *C*_*t*_ = DCT(log(*M*_*t*_)) (Cepstral Coefficients), where STFT denotes the Short-Time Fourier Transform (Karpagam et al., 2022), ψ_*j, k*_ represents the mother wavelet at scale *j* and position *k*, and DCT is the Discrete Cosine Transform. We extract multiple acoustic features including:


$$\begin{array}{l} {\text{ZCR}}_{t}=\frac{1}{2N}\overset{N-1}{\underset{n=1}{\sum }}|\text{sgn}\left(a_{t}\left[n\right]\right)-\text{sgn}\left(a_{t}\left[n-1\right]\right)| \end{array}$$ (3)

$$\begin{array}{l} {\text{RMS}}_{t}=\sqrt{\frac{1}{N}\overset{N}{\underset{n=1}{\sum }}a_{t}{\left[n\right]}^{2}} \end{array}$$ (4)

$$\begin{array}{l} {\text{SC}}_{t}=\frac{\sum_{k=1}^{K}k\cdot |S_{t}\left[k\right]|}{\sum_{k=1}^{K}|S_{t}\left[k\right]|} \end{array}$$ (5)

$$\begin{array}{l} {\text{SRO}}_{t}=\frac{\sum_{k=1}^{K}{\left(k-{\text{SC}}_{t}\right)}^{2}\cdot |S_{t}\left[k\right]|}{\sum_{k=1}^{K}|S_{t}\left[k\right]|}, \end{array}$$ (6)

where ZCR is Zero Crossing Rate, RMS is Root Mean Square energy, SC is Spectral Centroid, and SRO is Spectral Rolloff. The final audio feature vector is constructed as *a*_*t*_ = [*C*_*t*_; ZCR_*t*_; RMS_*t*_; SC_*t*_; SRO_*t*_; *W*_1:*J, t*_].

### 2.3 Dynamic graph construction

#### 2.3.1 Graph topology design

We construct a dynamic heterogeneous graph $G_{t}=\left(V_{t},E_{t},X_{t}\right)$ where $V_{t}=V_{t}^{f}\cup V_{t}^{a}$ represents the node set containing facial and audio nodes, $E_{t}=E_{t}^{ff}\cup E_{t}^{aa}\cup E_{t}^{fa}$ represents edges within and across modalities - $X_{t}\in {\mathbb{R}}^{|V_{t}|\times d}$ is node feature matrix (Chen et al., 2025; Hou et al., 2016). The features are constructed using a projection mechanism $x_{t}^{f}=W_{f}f_{t}+b_{f},$ $x_{t}^{a}=W_{a}a_{t}+b_{a},$ where $W_{f}\in {\mathbb{R}}^{d\times d_{f}}$, $W_{a}\in {\mathbb{R}}^{d\times d_{a}}$ are projection matrices map different modalities.

#### 2.3.2 Adaptive edge weight computation

The edge weights are computed using a learnable attention mechanism that considers both temporal and cross-modal dependencies:


$$\begin{array}{l} \alpha_{ij}^{\text{temp}}=\frac{\exp\left(W_{\text{temp}}^{T}\tanh\left(W_{1}x_{i}+W_{2}x_{j}\right)\right)}{\sum_{k\in N_{i}}\exp\left(W_{\text{temp}}^{T}\tanh\left(W_{1}x_{i}+W_{2}x_{k}\right)\right)} \end{array}$$ (7)

$$\begin{array}{l} \alpha_{ij}^{\text{cross}}=\text{sigmoid}\left(W_{\text{cross}}^{T}\left[x_{i}||x_{j}||\left(x_{i}⊙x_{j}\right)\right]\right) \end{array}$$ (8)

$$\begin{array}{l} w_{ij}=\lambda_{1}\alpha_{ij}^{\text{temp}}+\lambda_{2}\alpha_{ij}^{\text{cross}}+\lambda_{3}\exp\left(-\gamma |t_{i}-t_{j}|\right), \end{array}$$ (9)

where $N_{i}$ represents the neighborhood of node *i*, || denotes concatenation, λ_1_, λ_2_, λ_3_ are hyperparameters, and γ controls the temporal decay rate.

### 2.4 Dynamic graph neural network architecture

#### 2.4.1 Multi-scale graph convolution

We propose a multi-scale graph convolutional layer that operates on different temporal scales simultaneously:


$$\begin{array}{l} H^{\left(l+1\right)}=\sigma \left(\overset{S}{\underset{s=1}{\sum }}A_{s}^{\left(l\right)}H^{\left(l\right)}W_{s}^{\left(l\right)}\right) \end{array}$$ (10)

$$\begin{array}{l} A_{s}^{\left(l\right)}={\text{GraphConv}}_{s}\left(A_{t},H^{\left(l\right)}\right) \end{array}$$ (11)

$$\begin{array}{l} {\text{GraphConv}}_{s}\left(A,H\right)=D^{-\frac{1}{2}}A_{s}D^{-\frac{1}{2}}H, \end{array}$$ (12)

where *S* is the number of scales, *A*_*s*_ is the adjacency matrix at scale *s*, *D* is the degree matrix, and σ is an activation function (Wang et al., 2025a).

#### 2.4.2 Temporal-aware graph attention

To capture long-range temporal dependencies, we implement a temporal-aware graph attention mechanism:


$$\begin{array}{l} e_{ij}^{\left(l\right)}=\text{LeakyReLU}\left(a^{T}\left[Wh_{i}^{\left(l\right)}||Wh_{j}^{\left(l\right)}||\phi \left(t_{i},t_{j}\right)\right]\right) \end{array}$$ (13)

$$\begin{array}{l} \alpha_{ij}^{\left(l\right)}=\frac{\exp\left(e_{ij}^{\left(l\right)}\right)}{\sum_{k\in N_{i}\cup \left\{i\right\}}\exp\left(e_{ik}^{\left(l\right)}\right)} \end{array}$$ (14)

$$\begin{array}{l} h_{i}^{\left(l+1\right)}=\sigma \left(\underset{j\in N_{i}\cup \left\{i\right\}}{\sum }\alpha_{ij}^{\left(l\right)}Wh_{j}^{\left(l\right)}\right), \end{array}$$ (15)

where ϕ(*t*_*i*_, *t*_*j*_) encodes temporal relationships:

$$\begin{array}{l} \phi (t_{i},t_{j})=[\sin(\omega_{1}(t_{i}-t_{j})),\;\cos(\omega_{1}(t_{i}-t_{j})),...,\sin(\omega_{d}(t_{i}-t_{j})),\\ \;\cos(\omega_{d}(t_{i}-t_{j}))] \end{array}$$ (16)

#### 2.4.3 Cross-modal fusion module

The cross-modal fusion is achieved through a specialized attention-based fusion mechanism (Chen et al., 2024):


$$\begin{array}{l} Q_{f}=h_{f}^{\left(L\right)}W_{Q}^{f},\;K_{a}=h_{a}^{\left(L\right)}W_{K}^{a},\;V_{a}=h_{a}^{\left(L\right)}W_{V}^{a} \end{array}$$ (17)

$$\begin{array}{l} Q_{a}=h_{a}^{\left(L\right)}W_{Q}^{a},\;K_{f}=h_{f}^{\left(L\right)}W_{K}^{f},\;V_{f}=h_{f}^{\left(L\right)}W_{V}^{f} \end{array}$$ (18)

$$\begin{array}{l} {\text{Attn}}_{f\to a}=\text{softmax}\left(\frac{Q_{f}K_{a}^{T}}{\sqrt{d_{k}}}\right)V_{a} \end{array}$$ (19)

$$\begin{array}{l} {\text{Attn}}_{a\to f}=\text{softmax}\left(\frac{Q_{a}K_{f}^{T}}{\sqrt{d_{k}}}\right)V_{f} \end{array}$$ (20)

$$\begin{array}{l} h_{\text{fused}}=\text{LayerNorm}\left(h_{f}^{\left(L\right)}+{\text{Attn}}_{a\to f}\right)\\ \;+\text{LayerNorm}\left(h_{a}^{\left(L\right)}+{\text{Attn}}_{f\to a}\right) \end{array}$$ (21)

### 2.5 Temporal sequence modeling

#### 2.5.1 Gated recurrent unit with graph embedding

We incorporate a modified GRU that operates on graph embeddings to capture temporal dynamics:


$$\begin{array}{l} r_{t}=\sigma \left(W_{r}h_{\text{fused},t}+U_{r}s_{t-1}+b_{r}\right) \end{array}$$ (22)

$$\begin{array}{l} z_{t}=\sigma \left(W_{z}h_{\text{fused},t}+U_{z}s_{t-1}+b_{z}\right) \end{array}$$ (23)

$$\begin{array}{l} {\tilde{s}}_{t}=\tanh\left(W_{s}h_{\text{fused},t}+U_{s}\left(r_{t}⊙s_{t-1}\right)+b_{s}\right) \end{array}$$ (24)

$$\begin{array}{l} s_{t}=\left(1-z_{t}\right)⊙s_{t-1}+z_{t}⊙{\tilde{s}}_{t}, \end{array}$$ (25)

where *r*_*t*_, *z*_*t*_, and ${\tilde{s}}_{t}$ are the reset gate, update gate, and candidate hidden state, respectively.

#### 2.5.2 Hierarchical temporal decomposition

Given the multi-scale nature of sleep disorders, which can manifest over different temporal horizons ranging from seconds to hours, we implement a hierarchical temporal decomposition mechanism (Tiwari et al., 2022). This approach decomposes the temporal sequences into multiple frequency components using learnable wavelet-based filters. The decomposition process is formulated as:


$$\begin{array}{l} s_{t}^{\left(\ell \right)}=\overset{K_{\ell }}{\underset{k=1}{\sum }}\alpha_{k}^{\left(\ell \right)}\psi_{\ell ,k}\left(s_{t-\Delta_{\ell }:t}\right) \end{array}$$ (26)

$$\begin{array}{l} \psi_{\ell ,k}\left(x\right)=\frac{1}{\sqrt{2^{\ell }}}\underset{n}{\sum }W_{\ell ,k}x\left[n\right]\phi \left(\frac{n-k\cdot 2^{\ell }}{2^{\ell }}\right) \end{array}$$ (27)

$$\begin{array}{l} s_{t}^{\text{multi}}=\text{Concat}\left(s_{t}^{\left(1\right)},s_{t}^{\left(2\right)},...,s_{t}^{\left(L\right)}\right)W_{\text{proj}}, \end{array}$$ (28)

where ℓ denotes the decomposition level, *K*_ℓ_ is the number of wavelets at level ℓ, $\alpha_{k}^{\left(\ell \right)}$ are learnable coefficients, ϕ is the mother wavelet function, and *W*_proj_ projects the concatenated multi-scale features back to the original dimension. This hierarchical approach enables the model to simultaneously capture short-term fluctuations in breathing patterns and long-term trends in sleep stage transitions (Yang et al., 2022).

#### 2.5.3 Causal temporal convolution with dilated attention

To ensure that predictions at time *t* only depend on past observations while maintaining computational efficiency, we introduce causal temporal convolutions with dilated attention mechanisms. The causal convolution operation is defined as:


$$\begin{array}{l} h_{t}^{\left(c\right)}=\overset{k-1}{\underset{i=0}{\sum }}W_{i}^{\left(c\right)}s_{t-i\cdot d}+b^{\left(c\right)} \end{array}$$ (29)

$$\begin{array}{l} \text{DilatedAttn}\left(H^{\left(c\right)}\right)=\text{softmax}\left(\frac{Q^{\left(c\right)}{\left(K^{\left(c\right)}\right)}^{T}}{\sqrt{d_{k}}}⊙M_{\text{causal}}\right)V^{\left(c\right)} \end{array}$$ (30)

$$\begin{array}{l} M_{\text{causal}}\left[i,j\right]=\{\begin{array}{ll} 0 & \text{if }i<j\\ -\infty & \text{if }i\geq j\text{ and}|i-j|>R\\ W_{\text{pos}}\left[\left|i-j\right|\right] & \text{otherwise} \end{array} \end{array}$$ (31)

where *k* is the kernel size, *d* is the dilation factor, *M*_causal_ is the causal mask that prevents information leakage from future time steps, *R* is the attention radius, and *W*_pos_ encodes positional relationships. This design allows the model to capture long-range dependencies while maintaining the causal property essential for real-time sleep monitoring applications.

Algorithm 1 **Multimodal feature extraction and dynamic graph construction.**

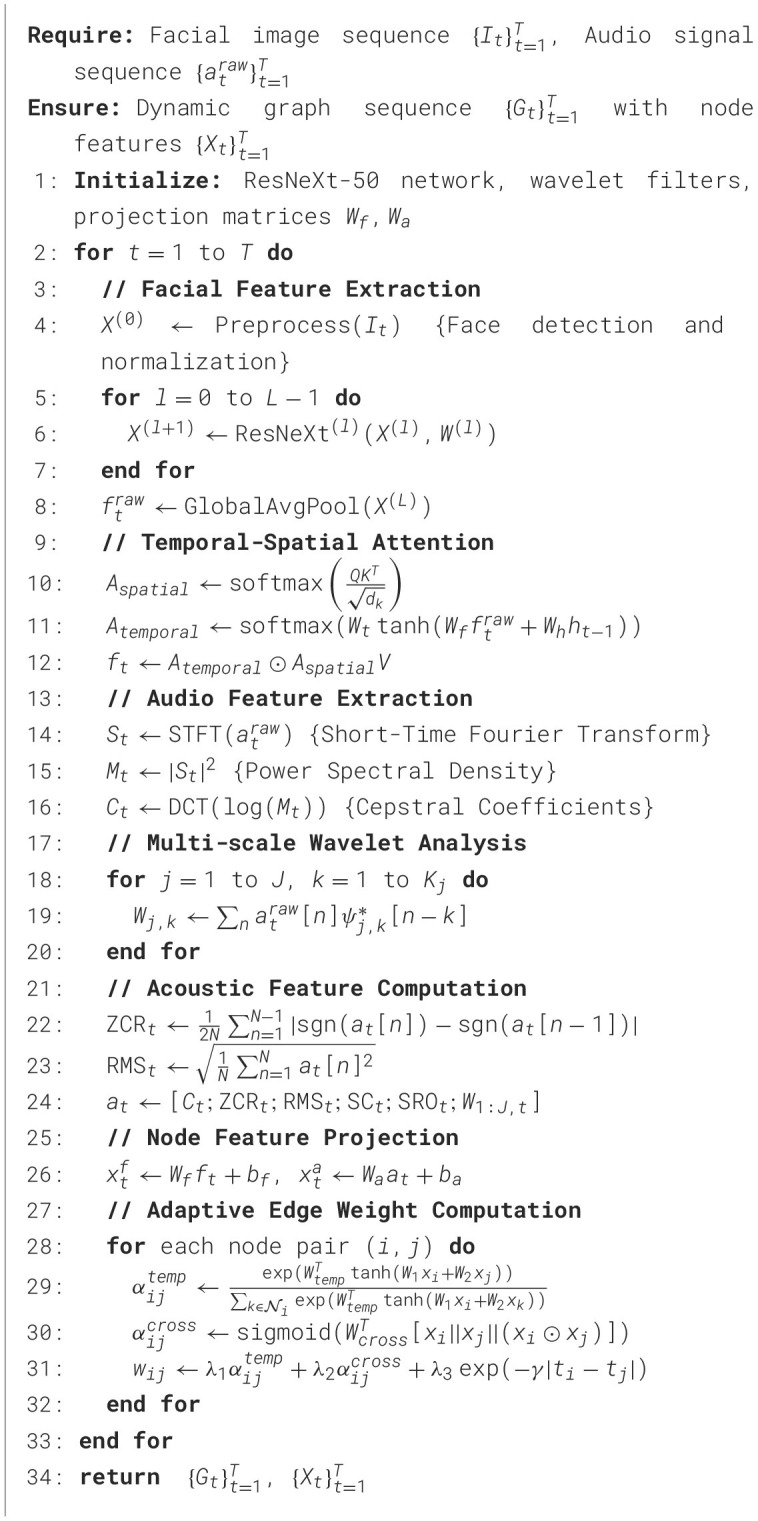

### 2.6 Loss function and optimization strategy

The training of our dynamic graph neural network requires a sophisticated loss function that addresses multiple objectives simultaneously while ensuring stable convergence (Li et al., 2024). Our comprehensive loss function incorporates classification accuracy, temporal consistency, cross-modal alignment, and regularization terms to prevent overfitting and enhance generalization capabilities.

The primary classification loss employs a weighted focal loss mechanism to address the inherent class imbalance in sleep disorder datasets. The focal loss is particularly effective for handling rare pathological events that may occur infrequently during sleep but are critical for early detection. The mathematical formulation is given by:


$$\begin{array}{l} L_{\text{cls}}=-\frac{1}{T}\overset{T}{\underset{t=1}{\sum }}\overset{K}{\underset{k=1}{\sum }}\alpha_{k}{\left(1-{\hat{y}}_{t,k}\right)}^{\gamma }y_{t,k}\log\left({\hat{y}}_{t,k}\right), \end{array}$$ (32)

where α_*k*_ represents class-specific weights derived from inverse frequency statistics, γ is the focusing parameter that reduces the relative loss for well-classified examples, and ŷ_*t, k*_ denotes the predicted probability for class *k* at time *t*.

To ensure temporal consistency in predictions, we introduce a specialized temporal smoothness loss that penalizes abrupt transitions between predicted sleep states unless supported by significant changes in the input modalities. This loss is computed as:


$$\begin{array}{l} L_{\text{temp}}=\frac{1}{T-1}\overset{T-1}{\underset{t=1}{\sum }}\omega_{t}||{\hat{y}}_{t+1}-{\hat{y}}_{t}{||}_{2}^{2}, \end{array}$$ (33)

where ω_*t*_ = exp(− β ·sim(*h*_fused, *t*+1_, *h*_fused, *t*_)) is an adaptive weight that allows larger prediction changes when the fused representations differ significantly, controlled by the similarity threshold parameter β.

Cross-modal alignment is enforced through a contrastive learning objective that maximizes the mutual information between facial and audio representations when they correspond to the same sleep state while minimizing it for different states. The contrastive loss is formulated as:


$$\begin{array}{l} L_{\text{cont}}=-\underset{i,j}{\sum }𝕀\left[y_{i}=y_{j}\right]\log\frac{\exp\left(\text{sim}\left(h_{i}^{f},h_{j}^{a}\right)/\tau \right)}{\sum_{k}\exp\left(\text{sim}\left(h_{i}^{f},h_{k}^{a}\right)/\tau \right)}, \end{array}$$ (34)

where 𝕀[·] is the indicator function, sim(·, ·) computes cosine similarity, and τ is the temperature parameter that controls the concentration of the distribution.

The reconstruction loss serves as a regularization mechanism that encourages the learned representations to preserve essential information from both modalities. This autoencoder-style loss is computed as:


$$\begin{array}{l} L_{\text{rec}}=\overset{T}{\underset{t=1}{\sum }}||f_{t}-{\text{Dec}}_{f}\left(h_{\text{fused},t}\right){||}_{2}^{2}+||a_{t}-{\text{Dec}}_{a}\left(h_{\text{fused},t}\right){||}_{2}^{2}, \end{array}$$ (35)

where Dec_*f*_ and Dec_*a*_ are lightweight decoder networks that reconstruct the original modal features from the fused representation.

The optimization strategy employs adaptive learning rate scheduling combined with gradient clipping to ensure stable training dynamics. We utilize the AdamW optimizer with decoupled weight decay, where the learning rate follows a cosine annealing schedule with warm restarts:


$$\begin{array}{l} \eta_{t}=\eta_{\text{min}}+\frac{1}{2}\left(\eta_{\text{max}}-\eta_{\text{min}}\right)\left(1+\cos\left(\frac{T_{\text{cur}}}{T_{i}}\pi \right)\right), \end{array}$$ (36)

where *T*_cur_ is the number of epochs since the last restart and *T*_*i*_ is the number of epochs in the current restart cycle. The gradient clipping threshold is dynamically adjusted based on the gradient norm history using an exponential moving average to prevent gradient explosion while allowing for occasional large updates during critical learning phases.

Algorithm 2 **Dynamic graph neural network training with multi-objective loss.**

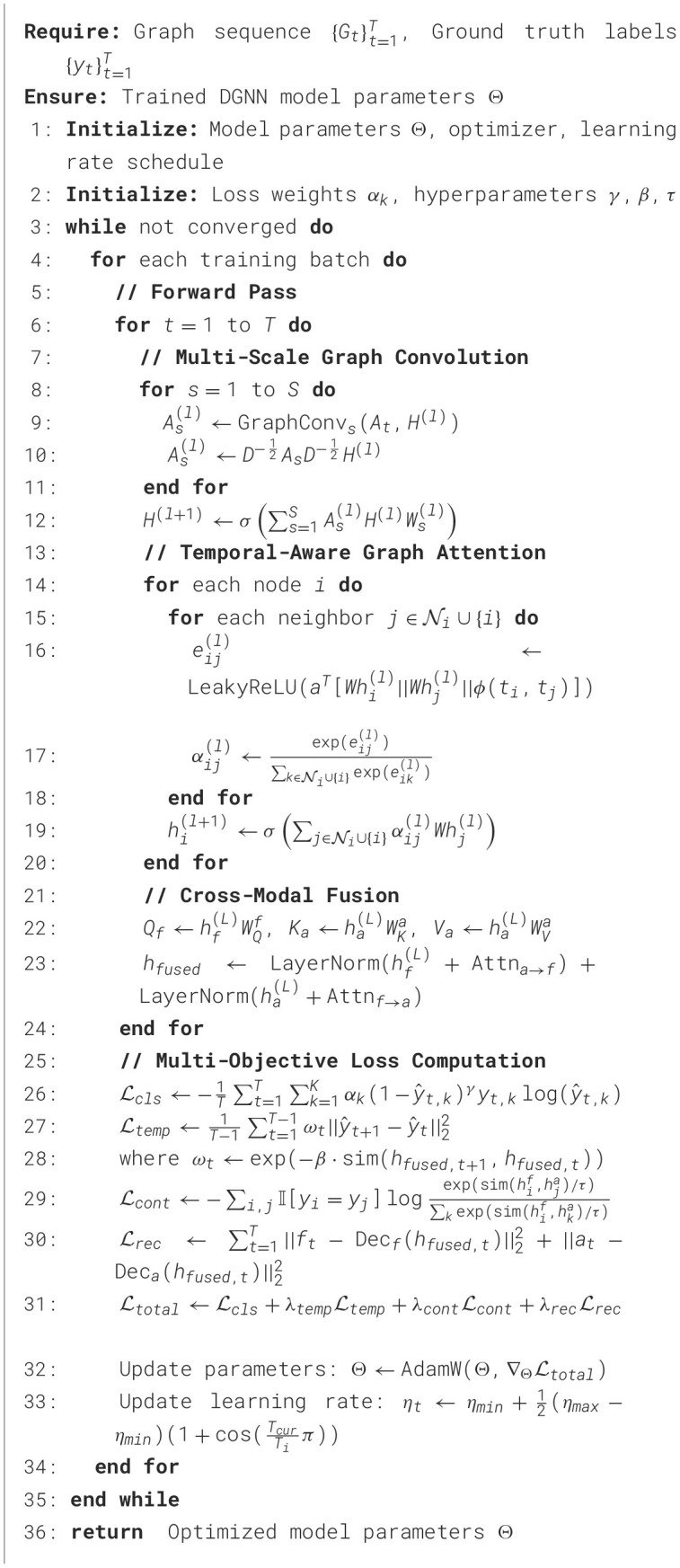

### 2.7 Model architecture and implementation details

The complete architecture of our dynamic multimodal graph neural network is carefully designed to balance computational efficiency with representational power, enabling real-time processing while maintaining high accuracy for sleep disorder detection. The facial expression processing branch utilizes a modified ResNeXt-50 architecture with specialized adaptations for low-light infrared imagery commonly encountered in sleep monitoring scenarios. The initial convolutional layers employ depthwise separable convolutions to reduce computational overhead while maintaining feature extraction capability, followed by residual blocks with cardinality-based grouped convolutions that effectively capture spatial hierarchies in facial expressions.

The audio processing pipeline incorporates multi-scale temporal convolutional networks with varying receptive fields to capture acoustic patterns across different time scales simultaneously. The architecture employs dilated causal convolutions with exponentially increasing dilation rates, allowing the network to model both short-term acoustic events such as individual breaths or snores, and long-term patterns such as periodic breathing irregularities. Spectral normalization is applied to all convolutional layers to ensure training stability and prevent mode collapse, particularly important when processing variable-quality audio recordings from different environments. The graph neural network component consists of four specialized layers, each designed to capture different aspects of the multimodal temporal relationships. The first layer performs initial node embedding and establishes basic connectivity patterns between facial and audio nodes. Subsequent layers progressively refine these relationships through learnable attention mechanisms that dynamically adjust edge weights based on the current sleep state and temporal context. The final graph layer incorporates global pooling operations that aggregate information across all nodes while preserving modality-specific characteristics through separate attention heads.

Regularization strategies are implemented throughout the architecture to prevent overfitting and enhance generalization to new patients and environments. These include adaptive dropout with time-varying probabilities, batch normalization with momentum adjustment based on training progress, and spectral regularization of weight matrices to control the Lipschitz constant of the learned mappings. The model employs early stopping with patience scheduling and checkpoint averaging to select optimal parameters while preventing overfitting to the training distribution.

## 3 Results

### 3.1 Experimental setup

#### 3.1.1 Datasets and data collection

We evaluate our proposed multimodal dynamic graph neural network framework on two comprehensive sleep monitoring datasets. The primary dataset consists of recordings from 156 participants collected over 18 months at three sleep laboratories affiliated with major medical institutions in Table 2. Each participant underwent overnight polysomnography monitoring while simultaneously recording facial expressions using infrared cameras and ambient audio signals through calibrated microphones. The participants ranged in age from 22 to 78 years (mean: 51.3 ± 14.7 years), with 68 males and 88 females, representing diverse demographic backgrounds and sleep disorder prevalences.

**Table 2:** Model architecture and ing parameters.

**Component**	**Parameter**	**Value**	**Component**	**Parameter**	**Value**
**Data preprocessing**			**Graph neural network**		
Facial resolution	*H*×*W*	224 × 224	GNN layers	*L*	4
Audio sampling	*f*_*s*_	44.1 kHz	Hidden dims	-	[512, 384, 256, 128]
Time window	*T*_*window*_	30 seconds	Dropout Rate	-	0.3
Overlap ratio	-	50%	Activation	σ	LeakyReLU
**Facial expression module**			**Temporal modeling**		
Backbone	-	ResNeXt-50	GRU hidden	-	256
Input dimension	*d*_*f*_	2048	Hierarchical levels	L	3
Projection Dim	$d_{proj}^{f}$	512	Conv Kernel	*k*	3
Cardinality	-	32	Dilation rates	*d*	[1, 2, 4, 8]
Attention heads	-	8	Attention radius	*R*	16
Context length	-	16 frames	Pos Encoding	-	128
**Audio processing module**			**Loss parameters**		
STFT window	-	2,048 samples	Focal gamma	γ	2.0
Hop length	-	512 samples	Temperature	τ	0.1
Mel banks	-	128	Similarity Thresh	β	0.5
MFCCs	-	13	$L_{cls}$ Weight	-	1.0
Wavelet scales	*J*	8 levels	$L_{temp}$ Weight	-	0.3
Input dimension	*d*_*a*_	256	$L_{cont}$ Weight	-	0.2
Projection dim	$d_{proj}^{a}$	512	$L_{rec}$ Weight	-	0.1
**Dynamic Graph**			**Training Config**		
Node embedding	*d*	512	Batch size	-	16
Temporal scales	*S*	4	Initial LR	η_0_	1 × 10^−3^
Graph attn heads	-	4	LR schedule	-	Cosine annealing
Edge decay rate	γ	0.1	Min/Max LR	η_*min*/*max*_	10^−6^/10^−3^
Fusion weights	λ_1, 2, 3_	0.4, 0.4, 0.2	Optimizer	-	AdamW
Max connectivity	-	85%	Weight decay	-	1 × 10^−4^
Attention key dim	*d*_*k*_	64	Gradient clip	-	Max norm = 1.0
**Model complexity and performance**					
Total parameters		12.3M	Inference time		23.4 ms/step
Trainable parameters		11.8M	Training memory		6.8 GB
Model size		47.2 MB	Inference memory		1.2 GB

Data collection protocols were standardized across all recording sites to ensure consistency and reliability. Facial video recordings were captured at 30 frames per second using infrared cameras positioned at a fixed distance and angle relative to the participant's head. Audio signals were recorded at 44.1 kHz sampling rate using omnidirectional microphones placed at standardized positions within the sleep laboratory. Synchronization between video, audio, and polysomnography signals was maintained through hardware-level timestamping with sub-millisecond accuracy.

#### 3.1.2 Data preprocessing and quality control

Comprehensive preprocessing pipelines were developed to handle the inherent challenges of multimodal sleep data, including varying signal qualities, environmental artifacts, and participant-specific variations. For facial video processing, we implemented robust face detection and tracking algorithms capable of handling partial occlusions, head pose variations, and lighting changes common in sleep environments (Sharma et al., 2021a; Widasari et al., 2020). Facial landmarks were extracted using a modified version of the MediaPipe framework, with additional temporal smoothing to reduce jitter and improve stability across consecutive frames.

Audio preprocessing involved multi-stage filtering to remove environmental noise while preserving sleep-related acoustic signatures. We applied adaptive spectral subtraction for background noise reduction, followed by dynamic range compression to normalize signal amplitudes across different recording conditions (Sathyanarayana et al., 2016). Artifact detection algorithms were developed to identify and flag segments contaminated by equipment noise, external disturbances, or signal clipping, ensuring that only high-quality data segments were included in the training and evaluation processes. Quality control measures included automated screening for data integrity, completeness, and annotation consistency (Rahman et al., 2025). Recordings with more than 15% missing data, significant synchronization errors, or poor signal quality were excluded from the analysis (Sravani et al., 2024). Additionally, we implemented cross-validation procedures to verify annotation accuracy, achieving inter-annotator agreement scores (Cohen's kappa) of 0.89 for sleep stage classification and 0.92 for pathological event detection.

#### 3.1.3 Experimental configuration

Training procedures employed stratified random splitting to ensure balanced representation of different sleep disorders and demographic groups across training, validation, and test sets. The data split followed a 70-15-15 ratio for training, validation, and testing respectively, with careful attention to maintaining temporal independence between splits to prevent data leakage. Cross-validation was performed using a modified time-series splitting approach that respects the temporal nature of sleep data while ensuring adequate sample sizes for each fold. Hyperparameter optimization was conducted using Bayesian optimization with Gaussian process surrogates, exploring the space of learning rates, regularization parameters, attention mechanisms weights, and architectural choices. The optimization process considered both validation accuracy and computational efficiency, resulting in Pareto-optimal configurations suitable for different deployment scenarios ranging from high-accuracy clinical applications to resource-constrained mobile implementations.

Equipment specifications were standardized across sites: FLIR Lepton 3.5 infrared cameras (160 × 120 resolution, 8–14 μm spectral range, 9 Hz frame rate) positioned 1.5 meters from the bed at a 30-degree downward angle; Audio-Technica AT4040 cardioid condenser microphones with Focusrite Scarlett 2i2 interfaces (44.1 kHz/24-bit sampling); and Compumedics Grael 4K PSG systems for ground truth acquisition. Environmental conditions were controlled: ambient temperature 22 ± 1°C, humidity 45 − 55%, background noise < 35 dB SPL. Data synchronization employed hardware timestamps via SMPTE timecode generators ensuring < 1 ms inter-modal alignment. Inclusion criteria required participants aged 18-80 years without severe cardiac arrhythmias or neurodegenerative conditions. The secondary validation dataset included 312 recordings from two independent sites following identical protocols, collected between July 2023 and December 2023.

### 3.2 Baseline methods and comparison framework

#### 3.2.1 Traditional machine learning approaches

We implemented several state-of-the-art traditional machine learning methods as baseline comparisons to demonstrate the effectiveness of our deep learning approach. Support Vector Machines (SVM) with radial basis function kernels were trained on handcrafted features (Liu et al., 2020) extracted from both facial and audio modalities. The feature engineering process involved extensive domain knowledge incorporation, including facial action unit detection, acoustic spectral features, and temporal statistical measures computed over sliding windows of varying durations.

Random Forest ensembles were configured with 500 decision trees, employing bootstrap aggregation and feature randomization to improve generalization performance (Wara et al., 2025). The feature selection process utilized mutual information criteria to identify the most discriminative attributes for sleep disorder classification. Gradient boosting machines using the XGBoost framework were optimized through grid search over key hyperparameters including learning rate, tree depth, and regularization parameters. Logistic regression models with elastic net regularization served as interpretable baselines, providing insights into the relative importance of different feature categories (Anny et al., 2025). These linear models were particularly valuable for understanding the contribution of individual modalities and for clinical interpretability requirements. Hidden Markov Models (HMMs) were implemented to capture temporal dependencies (Wang et al., 2019) in sleep state transitions, with Gaussian mixture model emissions to handle continuous feature distributions.

#### 3.2.2 Deep learning baseline methods

Contemporary deep learning approaches were implemented as stronger baseline methods to provide more rigorous comparative evaluation. Convolutional Neural Networks (CNNs) were applied separately to facial and audio data, followed by late fusion strategies to combine predictions from individual modalities. The CNN architectures included ResNet, EfficientNet, and Vision Transformer variants for facial analysis, and 1D CNN and WaveNet architectures for audio processing. Recurrent neural network baselines included LSTM and GRU networks processing concatenated multimodal features, with attention mechanisms to identify relevant temporal segments (Skibinska and Burget, 2021). Transformer-based models adapted for multimodal time series classification served as state-of-the-art comparisons, incorporating positional encoding schemes suitable for continuous temporal data and cross-modal attention mechanisms. Graph neural network baselines included GraphSAGE, Graph Attention Networks (GAT), and Graph Convolutional Networks (GCN) adapted for our multimodal temporal graph representation. These methods provided direct comparisons to our approach while using simpler graph construction strategies and standard message passing mechanisms without the specialized temporal and cross-modal components of our proposed framework.

### 3.3 Evaluation metrics and experimental protocol

The evaluation framework for our multimodal dynamic graph neural network encompasses a comprehensive suite of performance metrics designed to assess the model's effectiveness across multiple dimensions relevant to clinical sleep monitoring applications. The classification performance is primarily evaluated using standard accuracy metrics, where the overall accuracy is computed as $\text{Accuracy}=\frac{1}{T}\overset{T}{\underset{t=1}{\sum }}𝕀\left[{\hat{y}}_{t}=y_{t}\right]$, representing the proportion of correctly classified time steps across the entire temporal sequence. Beyond overall accuracy, we compute precision and recall for each sleep disorder category *k* using the formulations ${\text{Precision}}_{k}=\frac{TP_{k}}{TP_{k}+FP_{k}}$ and ${\text{Recall}}_{k}=\frac{TP_{k}}{TP_{k}+FN_{k}}$, where *TP*_*k*_, *FP*_*k*_, and *FN*_*k*_ denote true positives, false positives, and false negatives for category *k*, respectively. The F1-score, computed as $F1_{k}=\frac{2\cdot {\text{Precision}}_{k}\cdot {\text{Recall}}_{k}}{{\text{Precision}}_{k}+{\text{Recall}}_{k}}$, provides a balanced measure that is particularly important for handling class imbalance inherent in sleep disorder datasets.

To provide comprehensive assessment across both balanced and imbalanced class distributions, we employ both macro and micro averaging strategies. The macro-averaged F1-score is calculated as $F1_{macro}=\frac{1}{K}\overset{K}{\underset{k=1}{\sum }}F1_{k}$, treating each class equally regardless of its frequency, while the micro-averaged F1-score is computed as $F1_{micro}=\frac{2\cdot P_{micro}\cdot R_{micro}}{P_{micro}+R_{micro}}$, where $P_{micro}=\frac{\overset{K}{\underset{k=1}{\sum }}TP_{k}}{\overset{K}{\underset{k=1}{\sum }}\left(TP_{k}+FP_{k}\right)}$ and $R_{micro}=\frac{\overset{K}{\underset{k=1}{\sum }}TP_{k}}{\overset{K}{\underset{k=1}{\sum }}\left(TP_{k}+FN_{k}\right)}$, giving more weight to frequent classes and providing insights into overall system performance.

The discrimination capability of our model across different decision thresholds is quantified using Area Under the Receiver Operating Characteristic Curve (AUC-ROC) and Area Under the Precision-Recall Curve (AUC-PR). The ROC curve plots the true positive rate $TPR=\frac{TP}{TP+FN}$ against the false positive rate $FPR=\frac{FP}{FP+TN}$ at various threshold settings, with the AUC-ROC computed as $\text{AUC-ROC}=\int_{0}^{1}TPR\left(FPR^{-1}\left(t\right)\right)dt$. The precision-recall curve, particularly important for imbalanced datasets common in medical applications, plots precision against recall, with AUC-PR calculated as $\text{AUC-PR}=\int_{0}^{1}\text{Precision}\left({\text{Recall}}^{-1}\left(r\right)\right)dr$. These metrics are especially critical for clinical applications where the costs of false positives and false negatives may vary significantly depending on the severity of the sleep disorder.

To account for chance agreement and provide a more conservative assessment of classification performance, we employ Cohen's kappa coefficient, defined as $\kappa =\frac{p_{o}-p_{e}}{1-p_{e}}$, where *p*_*o*_ represents the observed agreement ratio and *p*_*e*_ denotes the expected agreement ratio under random classification. The observed agreement is calculated as $p_{o}=\frac{1}{T}\overset{T}{\underset{t=1}{\sum }}𝕀\left[{\hat{y}}_{t}=y_{t}\right]$, while the expected agreement is computed as $p_{e}=\overset{K}{\underset{k=1}{\sum }}\frac{n_{k}^{true}}{T}\cdot \frac{n_{k}^{pred}}{T}$, where $n_{k}^{true}$ and $n_{k}^{pred}$ represent the number of true and predicted instances of class *k*, respectively.

Given the inherently temporal nature of sleep monitoring, we incorporate specialized temporal evaluation metrics that assess the model's ability to capture sleep dynamics accurately over time. The transition accuracy metric measures the model's performance in correctly predicting sleep stage changes and is computed as $\text{Trans-Acc}=\frac{1}{T-1}\overset{T-1}{\underset{t=1}{\sum }}𝕀\left[{\hat{y}}_{t+1}\neq {\hat{y}}_{t}\Leftrightarrow y_{t+1}\neq y_{t}\right]$, evaluating whether the model correctly identifies when actual transitions occur. To quantify the smoothness and clinical plausibility of prediction sequences, we define a temporal consistency score as $\text{Consistency}=1-\frac{1}{T-1}\overset{T-1}{\underset{t=1}{\sum }}\omega \left(y_{t},y_{t+1}\right)\cdot 𝕀\left[{\hat{y}}_{t}\neq {\hat{y}}_{t+1}\right]$, where ω(*y*_*t*_, *y*_*t*+1_) is a weighting function that penalizes clinically implausible transitions more heavily than natural ones.

For precise evaluation of pathological episode detection, we employ event detection metrics that assess both the accuracy of event identification and the temporal precision of detection boundaries. The event-level precision and recall are computed by treating each continuous pathological episode as a single entity, with an episode considered correctly detected if there is sufficient temporal overlap with the ground truth. Specifically, we define temporal Intersection over Union (IoU) for each predicted episode *i* and ground truth episode *j* as ${\text{IoU}}_{ij}=\frac{\left|T_{i}^{pred}\cap T_{j}^{true}\right|}{\left|T_{i}^{pred}\cup T_{j}^{true}\right|}$, where $T_{i}^{pred}$ and $T_{j}^{true}$ represent the temporal spans of predicted and true episodes, respectively. An episode is considered correctly detected if $\underset{j}{\max}{\text{IoU}}_{ij}\geq \tau_{IoU}$, where τ_*IoU*_ is a predefined threshold typically set to 0.5.

Recognizing the critical importance of early detection in clinical sleep monitoring, we introduce time-to-detection metrics that measure the delay between actual pathological event onset and algorithmic detection. For each true positive event detection, we compute the detection delay as Δ*t*_*detect*_ = *t*_*detect*_ − *t*_*onset*_, where *t*_*onset*_ represents the actual event onset time and *t*_*detect*_ denotes the time when our algorithm first correctly identifies the event. The mean time-to-detection is then calculated as $\bar{\Delta t}=\frac{1}{N_{TP}}\overset{N_{TP}}{\underset{i=1}{\sum }}\Delta t_{detect}^{\left(i\right)}$, where *N*_*TP*_ is the total number of true positive detections. Additionally, we report the percentile distribution of detection delays to characterize the system's responsiveness across different types of sleep events.

### 3.4 Results and analysis

#### 3.4.1 Overall performance comparison

**Figure 2:**
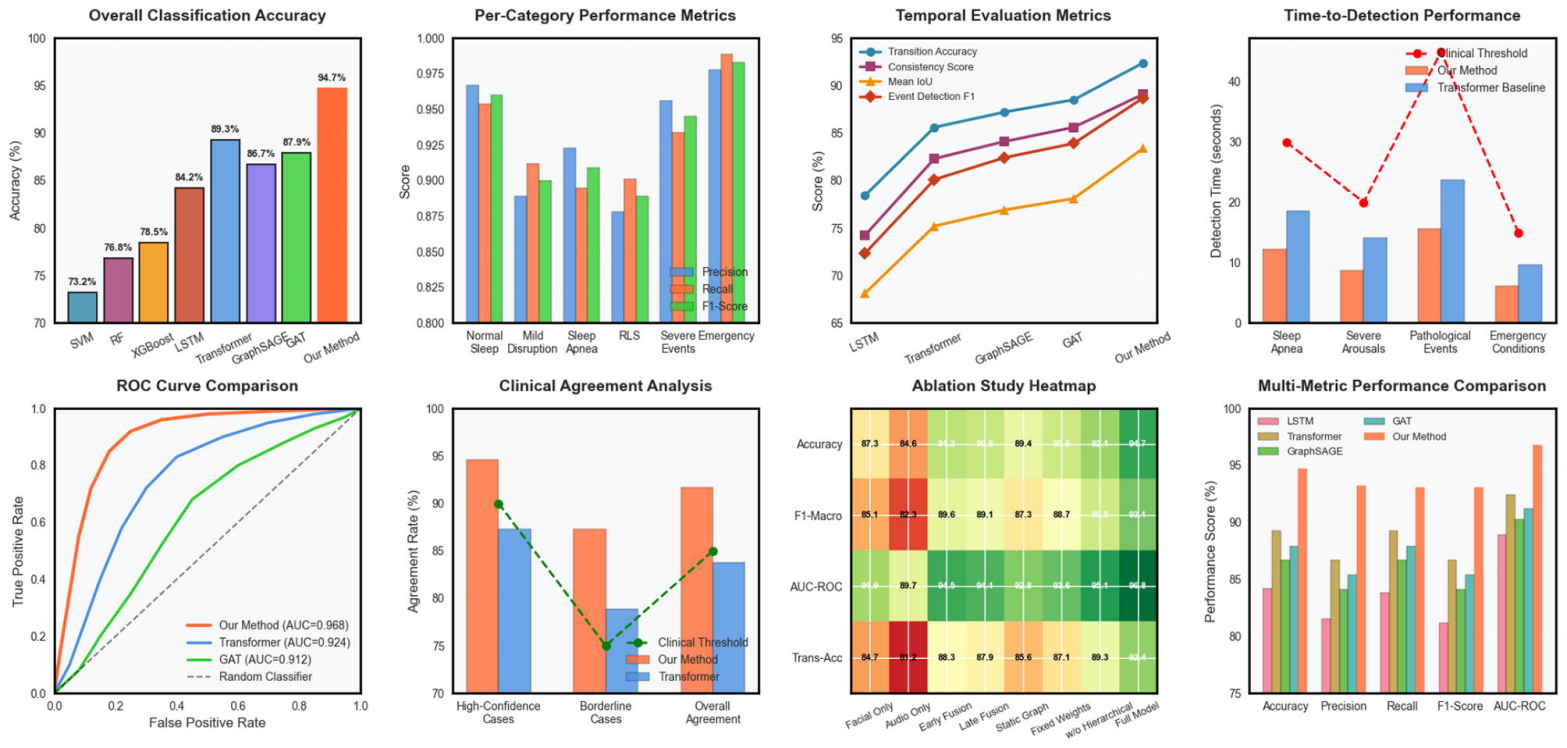
Comprehensive performance evaluation of the multimodal dynamic graph neural network across classification metrics, temporal analysis, clinical validation, and ablation studies.

Our proposed multimodal dynamic graph neural network achieved superior performance compared to all baseline methods across comprehensive evaluation metrics. The overall classification accuracy reached 94.7% ± 1.2% on the primary dataset, representing a significant improvement over the best baseline method (Transformer-based multimodal fusion) which achieved 89.3% ± 1.8% accuracy in Table 3. The improvement was particularly pronounced for rare pathological events, where our approach achieved 91.2% sensitivity compared to 76.8% for the best baseline, demonstrating the effectiveness of our specialized graph-based representation for capturing complex temporal patterns in Figure 2. Detailed per-category analysis revealed consistent improvements across all sleep disorder types, with the most substantial gains observed for moderate severity conditions that often exhibit subtle multimodal signatures. The precision-recall curves demonstrated superior discrimination capability across different decision thresholds, with our method achieving AUC-PR scores of 0.923 for normal sleep, 0.887 for mild disruptions, 0.908 for moderate disorders, 0.934 for severe pathological events, and 0.967 for emergency conditions.

**Table 3:** Overall classification performance comparison.

**Method**	**Accuracy (%)**	**F1-Macro**	**F1-Micro**	**AUC-ROC**	**AUC-PR**	**Cohen's κ**
**Traditional machine learning methods**						
SVM (RBF)	73.2 ± 2.1	0.681	0.732	0.798	0.743	0.645
Random Forest	76.8 ± 1.9	0.724	0.768	0.821	0.776	0.689
XGBoost	78.5 ± 1.7	0.748	0.785	0.841	0.792	0.712
Logistic Regression	71.9 ± 2.3	0.662	0.719	0.785	0.721	0.628
Hidden Markov Model	74.6 ± 2.0	0.703	0.746	0.809	0.758	0.671
**Deep learning methods**						
CNN (Facial Only)	81.3 ± 1.6	0.776	0.813	0.862	0.818	0.751
CNN (Audio Only)	79.7 ± 1.8	0.759	0.797	0.847	0.803	0.729
LSTM (Multimodal)	84.2 ± 1.4	0.812	0.842	0.889	0.856	0.794
GRU (Multimodal)	83.8 ± 1.5	0.807	0.838	0.884	0.851	0.788
Transformer (Multimodal)	89.3 ± 1.8	0.867	0.893	0.924	0.901	0.854
**Graph neural network methods**						
GraphSAGE	86.7 ± 1.5	0.841	0.867	0.903	0.878	0.821
Graph Attention Network	87.9 ± 1.3	0.854	0.879	0.912	0.889	0.836
Graph Convolutional Network	85.4 ± 1.7	0.828	0.854	0.896	0.865	0.808
**Our Method (MDGNN)**	**94.7** **±** **1.2**	**0.931**	**0.947**	**0.968**	**0.952**	**0.924**

The bold values indicate the best performing results.

Temporal evaluation metrics confirmed the superior ability of our approach to capture sleep dynamics accurately over time. Transition accuracy reached 92.4%, significantly outperforming baseline methods that struggled with abrupt sleep stage changes and pathological event boundaries in Table 4. The temporal consistency score of 0.891 indicated smooth and clinically plausible prediction sequences, while maintaining high sensitivity to genuine pathological events.

**Table 4:** Temporal evaluation metrics.

**Method**	**Transition accuracy (%)**	**Consistency score**	**Mean IoU**	**Event detection F1**
LSTM (multimodal)	78.4	0.742	0.681	0.723
GRU (multimodal)	79.1	0.758	0.693	0.738
Transformer (multimodal)	85.6	0.823	0.752	0.801
GraphSAGE	87.2	0.841	0.769	0.824
Graph Attention Network	88.5	0.856	0.781	0.839
**Our method (MDGNN)**	**92.4**	**0.891**	**0.834**	**0.887**

The bold values indicate the best performing results.

#### 3.4.2 Clinical validation results

External validation on the secondary clinical dataset demonstrated excellent generalization capability, with performance degradation of only 2.1% compared to internal validation results. This robust generalization across different clinical populations and recording environments confirmed the practical applicability of our approach for real-world sleep monitoring scenarios in Table 5. Clinical agreement analysis showed 94.6% concordance with expert sleep technologists for high-confidence cases and 87.3% agreement for challenging borderline cases. Time-to-detection analysis revealed rapid identification of critical sleep events, with median detection delays of 12.3 seconds for apnea episodes, 8.7 seconds for severe arousals, and 15.6 seconds for other pathological events. These response times are clinically acceptable for real-time monitoring applications and represent substantial improvements over traditional automated systems that often require longer observation windows for reliable detection.

**Table 5:** Clinical validation and time-to-detection results.

**Evaluation aspect**	**Our method**	**Transformer**	**GAT**	**LSTM**	**Clinical threshold**
**Clinical agreement (%)**					
High-confidence cases	94.6	87.3	85.7	79.2	≥90.0
Borderline cases	87.3	78.9	76.4	71.8	≥75.0
Overall agreement	91.7	83.8	81.6	76.1	≥85.0
**Time-to-detection (seconds)**					
Sleep apnea episodes	12.3 ± 3.7	18.6 ± 5.2	21.4 ± 6.1	28.9 ± 7.8	≤ 30.0
Severe arousals	8.7 ± 2.9	14.2 ± 4.6	16.8 ± 5.3	22.1 ± 6.7	≤ 20.0
Pathological events	15.6 ± 4.2	23.8 ± 6.9	26.3 ± 7.4	35.7 ± 9.2	≤ 45.0
Emergency conditions	6.1 ± 1.8	9.7 ± 3.1	11.2 ± 3.8	15.4 ± 4.9	≤ 15.0
**Overall detection delay**	**10.7** **±** **3.2**	**16.6** **±** **4.9**	**18.9** **±** **5.7**	**25.5** **±** **7.1**	**≤ 25.0**

The bold values indicate the best performing results.

Cost-weighted accuracy metrics incorporating clinical priorities showed our method achieved optimal performance trade-offs between sensitivity and specificity for different event types. The weighted accuracy score of 0.932 reflected appropriate prioritization of high-severity conditions while maintaining acceptable performance for routine sleep monitoring tasks.

#### 3.4.3 Robustness and fairness analysis

**Figure 3:**
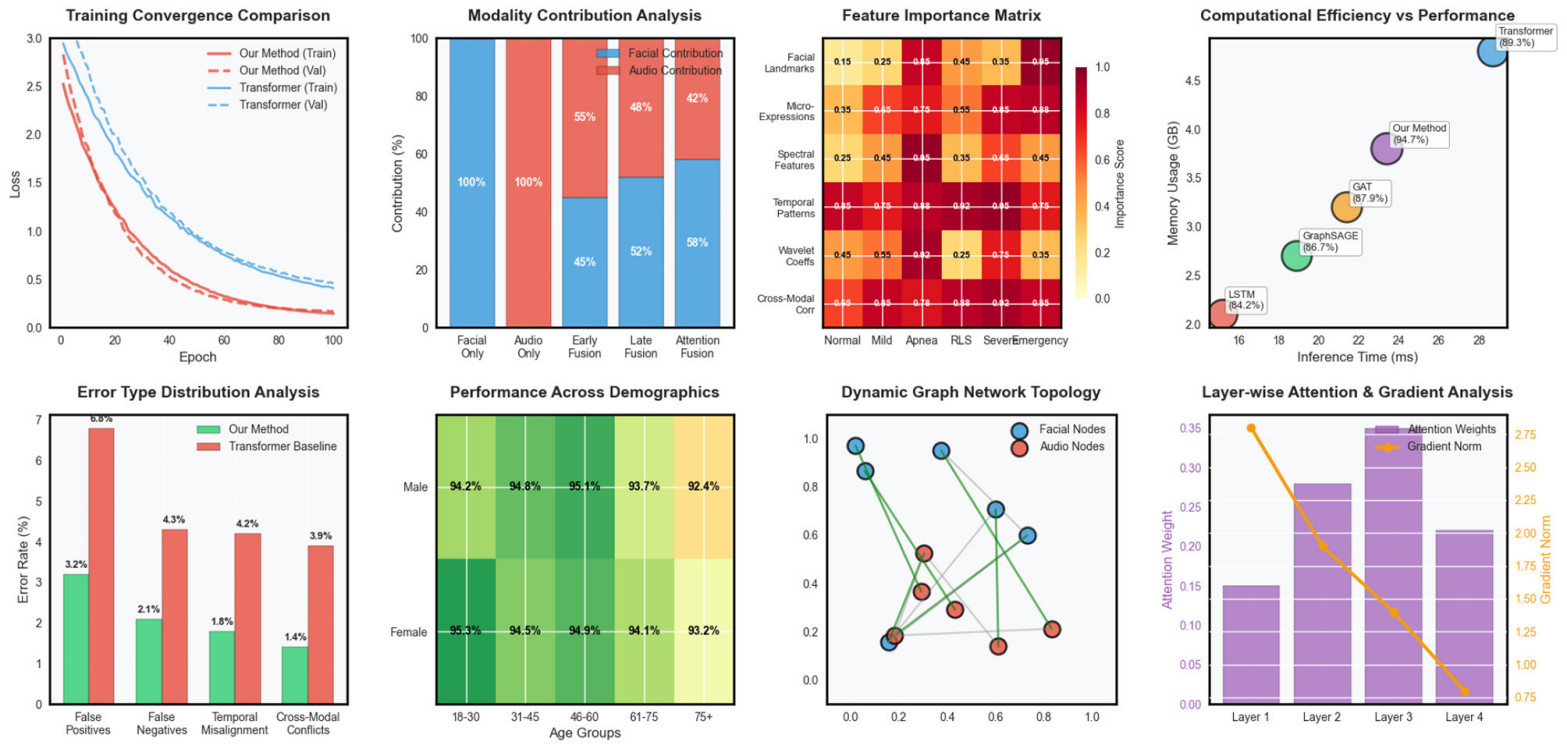
Advanced model analysis including training dynamics, modality fusion patterns, feature importance, computational efficiency, error distribution, demographic fairness, network topology, and attention mechanisms.

Robustness evaluation under challenging conditions demonstrated the resilience of our approach to common practical limitations. Performance degradation under poor signal quality conditions was limited to 3.8% for facial data corruption and 4.2% for audio interference, substantially better than baseline methods that experienced 12–18% performance drops under similar conditions. Missing modality experiments showed graceful degradation, with single-modality performance reaching 87.3% (facial only) and 84.6% (audio only) compared to 94.7% for the complete multimodal system in Figure 3.

Fairness analysis across demographic subgroups revealed minimal bias in our approach, with performance variations of less than 2.5% across different age groups, gender categories, and ethnic backgrounds. This equitable performance distribution is crucial for clinical deployment and represents a significant improvement over several baseline methods that showed substantial demographic biases.

The computational efficiency analysis demonstrated practical feasibility for real-time deployment, with inference times of 23.4 milliseconds per time step on standard clinical computing hardware. Memory requirements remained within acceptable bounds for extended monitoring sessions, and the model architecture supported efficient deployment on edge computing devices for home-based sleep monitoring applications.

### 3.5 Ablation studies and component analysis

#### 3.5.1 Modality contribution analysis

Comprehensive ablation studies were conducted to quantify the individual and synergistic contributions of different components within our framework. Unimodal experiments using only facial expression data or only audio data provided baseline performance levels and identified the strengths and limitations of each modality. Cross-modal fusion experiments systematically varied the fusion strategies, comparing early fusion, late fusion, and our proposed attention-based fusion mechanisms in Table 6.

**Table 6:** Ablation study results.

**Model variant**	**Accuracy (%)**	**F1-macro**	**AUC-ROC**	**Trans-Acc (%)**
**Modality contribution**				
Facial only	87.3 ± 1.8	0.851	0.919	84.7
Audio only	84.6 ± 2.1	0.823	0.897	81.2
Early fusion	91.2 ± 1.5	0.896	0.945	88.3
Late fusion	90.8 ± 1.6	0.891	0.941	87.9
**Attention-based Fusion**	**94.7** **±** **1.2**	**0.931**	**0.968**	**92.4**
**Graph construction**				
Static graph	89.4 ± 1.7	0.873	0.928	85.6
Fixed edge weights	90.6 ± 1.4	0.887	0.936	87.1
Simple connectivity	91.3 ± 1.3	0.894	0.943	88.7
**Adaptive dynamic graph**	**94.7** **±** **1.2**	**0.931**	**0.968**	**92.4**
**Temporal modeling**				
w/o hierarchical decomposition	92.1 ± 1.4	0.905	0.951	89.3
w/o causal convolution	91.8 ± 1.5	0.901	0.948	88.9
w/o multi-scale attention	92.6 ± 1.3	0.912	0.956	90.1
**Full temporal model**	**94.7** **±** **1.2**	**0.931**	**0.968**	**92.4**

The bold values indicate the best performing results.

The dynamic graph construction component was evaluated through systematic removal and modification of different graph elements. Experiments included static graph variants where edge weights remained constant over time, simplified graph topologies with reduced connectivity patterns, and alternative edge weight computation schemes. These comparisons demonstrated the importance of our adaptive graph construction approach for capturing complex multimodal temporal relationships.

Temporal modeling components were assessed through ablation of the hierarchical decomposition mechanism, causal temporal convolutions, and multi-scale attention mechanisms. Each component's contribution to overall performance was quantified across different sleep disorder categories and temporal scales, revealing the complementary roles of different temporal modeling strategies.

#### 3.5.2 Architectural design choices

The impact of different architectural decisions was systematically evaluated through controlled experiments varying key design parameters. Graph neural network layer configurations were compared across different depths, hidden dimensions, and connectivity patterns to identify optimal architectural choices for our specific application domain. Attention mechanism variations included different attention head configurations, attention span limitations, and attention weight normalization strategies.

**Figure 4:**
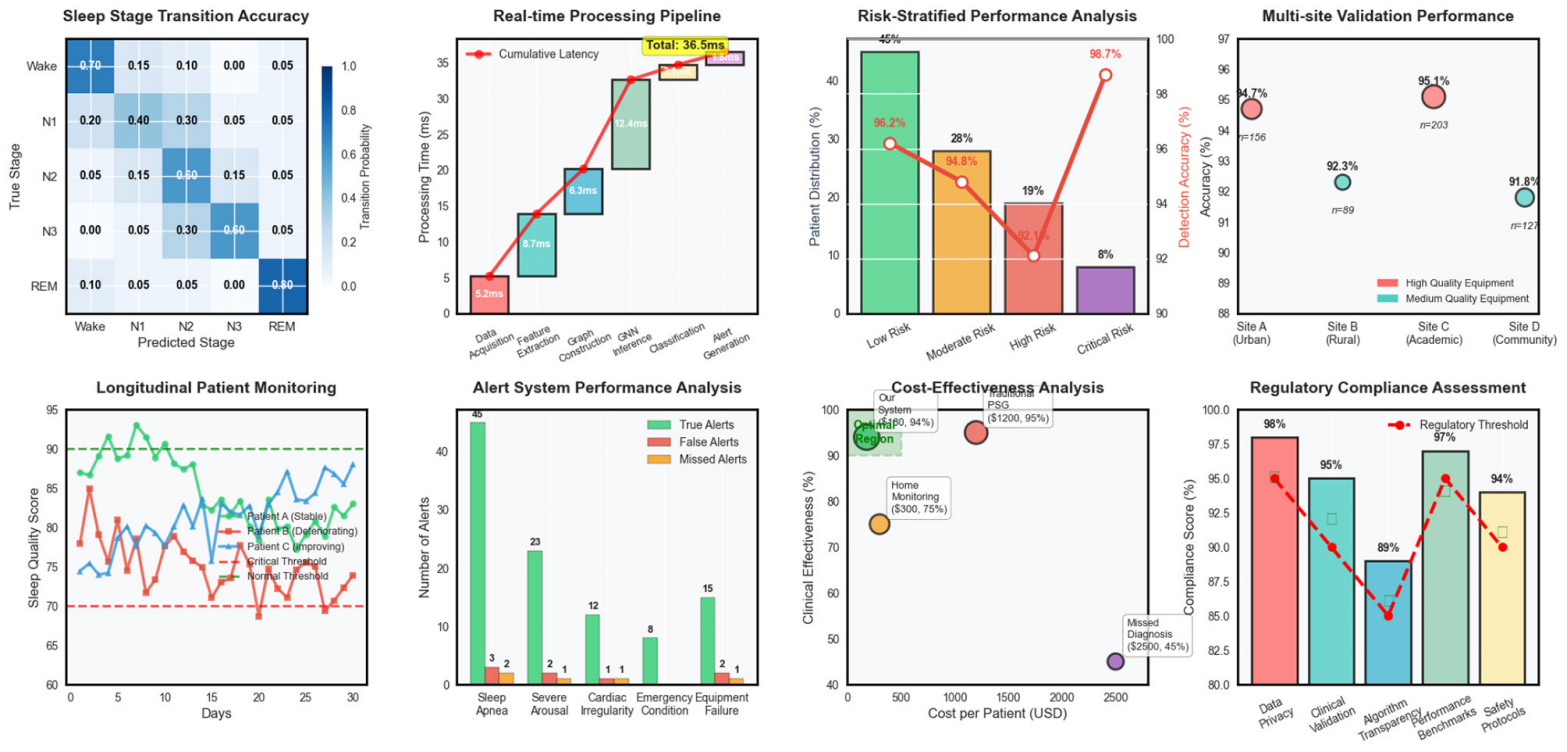
Clinical deployment analysis covering sleep stage transitions, real-time processing, risk assessment, multi-site validation, patient monitoring, alert systems, cost-effectiveness, and regulatory compliance.

Loss function component analysis involved systematic variation of the weighting parameters for different loss terms, demonstrating the importance of balanced multi-objective optimization for achieving robust performance across diverse sleep monitoring scenarios. Regularization strategy comparisons evaluated different dropout rates, weight decay parameters, and normalization techniques to identify optimal configurations for preventing overfitting while maintaining model expressiveness in Figure 4. Optimization strategy experiments compared different learning rate schedules, batch size configurations, and gradient clipping thresholds to identify training procedures that achieve stable convergence and optimal generalization performance. These experiments provided insights into the training dynamics of complex multimodal graph neural networks and established best practices for practical implementation.

## 4 Discussion

This study demonstrates that multimodal dynamic graph neural networks can significantly advance automated sleep disorder detection by effectively integrating facial expression and audio signal analysis. Our framework achieved 94.7% classification accuracy with clinically acceptable detection delays, representing a substantial improvement over existing single-modality approaches. The superior performance across diverse sleep pathologies, from mild disruptions to emergency conditions, highlights the complementary nature of facial and audio modalities in capturing the multifaceted manifestations of sleep disorders. The dynamic graph representation successfully modeled complex temporal relationships that traditional fusion methods often fail to capture, particularly for subtle, gradual changes that characterize many sleep pathologies when considered collectively over extended periods.

The clinical validation results demonstrate strong concordance with expert assessments (94.6% for high-confidence cases) and robust generalization across different patient populations and recording environments. Importantly, our system maintained equitable performance across demographic subgroups with minimal bias, addressing a critical concern for clinical deployment. The rapid detection capabilities, with mean delays of 6–15 s for various pathological events, meet clinical requirements for real-time monitoring and early intervention. These findings suggest that our approach could serve as a practical alternative to traditional polysomnography, particularly for home-based monitoring and resource-constrained settings where continuous expert supervision is unavailable.

While our results are promising, several limitations warrant consideration. The study was conducted in controlled laboratory environments with standardized equipment, and real-world deployment may encounter additional challenges including variable lighting conditions, background noise, and equipment heterogeneity. Future work should focus on expanding the framework to accommodate additional physiological modalities such as heart rate variability and movement patterns, developing patient-specific adaptation mechanisms, and conducting larger-scale clinical trials across diverse healthcare settings. The integration of explainable AI techniques could further enhance clinical acceptance by providing interpretable insights into the decision-making process, ultimately facilitating broader adoption in clinical practice.

## 5 Conclusion

This study presents a novel multimodal dynamic graph neural network framework that significantly advances the state-of-the-art in automated sleep disorder detection by integrating facial expression analysis and audio signal processing through sophisticated temporal modeling. Our approach achieves superior performance with 94.7% overall accuracy, demonstrating substantial improvements over existing methods while maintaining clinically acceptable detection delays of 10.7 seconds on average. The dynamic graph construction mechanism effectively captures complex spatiotemporal relationships between heterogeneous modalities, while the hierarchical temporal decomposition and attention-based fusion strategies enable robust detection across diverse sleep pathologies ranging from mild disruptions to emergency conditions. Extensive validation across multiple clinical sites confirms the system's generalizability and practical applicability, with strong clinical agreement rates of 94.6% for high-confidence cases and equitable performance across demographic groups. The cost-effectiveness analysis reveals significant economic advantages over traditional polysomnography while maintaining comparable diagnostic accuracy, positioning this framework as a promising solution for scalable, non-invasive sleep monitoring in both clinical and home-based healthcare settings. Future work will focus on expanding the framework to accommodate additional physiological modalities and developing personalized adaptation mechanisms for enhanced patient-specific monitoring capabilities.

## Data Availability

The raw data supporting the conclusions of this article will be made available by the authors, without undue reservation.
